# An unusual clinical presentation resembling superior vena cava syndrome post heart surgery

**DOI:** 10.1186/1476-7120-3-31

**Published:** 2005-10-03

**Authors:** Angel López-Candales, David Kaczorowski, Ronald Pellegrini

**Affiliations:** 1 Cardiothoracic Division, Department of Surgery and the Cardiovascular Institute*, University of Pittsburgh Medical Center, Pittsburgh, PA, USA

**Keywords:** Aortic valve mass, cardiac tamponade, heart surgery, patent foramen ovale, right ventricle, transesophageal echocardiography, tricuspid valve

## Abstract

**Background:**

An unusual sequence of post operative events heralded by hemodynamic deterioration followed by dyspnea and rapidly progressive dilatation of superficial neck and facial veins, resembling a superior vena cava syndrome, two days post surgical resection of filamentous aortic valve masses, closure of a patent foramen ovale, and performance of a modified Maze procedure for atrial fibrillation in a patient that presented with transient neurologic findings is presented.

**Case Presentation:**

Although both clinical findings and hemodynamic derangements completely resolved following tricuspid valve repair aimed to correct the new onset severe tricuspid regurgitation noted post operatively; a clear mechanism was not readily obvious and diagnostic testing data somewhat conflictive. We present a careful retrospective examination of all clinical data and review possible clinical entities that could have been implicated in this particular case and recognize that transesophageal echocardiographic findings were most useful in identifying the best course of action.

**Conclusion:**

After reviewing all clinical data and despite the inconclusive nature of test results; the retrospective examination of transesophageal echocardiographic findings proved to be most useful in identifying the best course of action. We postulate that in our case, resolution of the suspected pulmonary embolism with anticoagulation and reestablishment of a normal right ventricular geometry with tricuspid valve repair worked in unison in restoring normal hemodynamics and resolving both dyspnea and venous dilatation.

## Background

It has been suggested that when patients suffer unexpected findings or develop multiple complications after any intervention, adverse outcomes might be expected if a strategy is not promptly implemented to identify the offending or triggering mechanism(s). In this case we present a patient who underwent surgical resection of filamentous aortic valve masses, closure of a patent foramen ovale, and performance of a modified Maze procedure for atrial fibrillation who then developed a series of unusual post operative events heralded by hemodynamic deterioration, dyspnea and rapidly progressive dilatation of superficial neck and facial veins, resembling a superior vena cava syndrome; two days post surgery. A careful retrospective examination of all clinical data is presented with a detailed review of all possible clinical entities.

## Case Report

A 72-year-old woman with chronic atrial fibrillation, temporal arteritis, and avascular necrosis of the right hip requiring hemiarthroplasty that was complicated by deep venous thrombosis in the past, now presents with new onset left upper extremity clumsiness. A head computed tomography (CT) scan and subsequent magnetic resonance imaging study revealed a hemorrhage into the right parietal and occipital regions of the brain. In view of these findings, warfarin therapy was discontinued and as part of the workup a transesophageal echocardiogram (TEE) was performed and revealed a 4 × 6 mm mass on the aortic valve and a patent foramen ovale (PFO) with bidirectional shunting (right to left after the intravenous injection of agitated saline), mild bi-atrial enlargement, mildly dilated but normally functioning right ventricle, normal left ventricular systolic function and no significant valvular regurgitation. The patient was afebrile and blood cultures were negative. The patient was referred to our institution for surgical evaluation given the TEE findings and the patient clinical presentation. Surgical resection of the aortic valve mass and closure of the PFO were recommended.

Upon examination of the aortic valve, a filamentous mass was identified on the leading edge of the noncoronary cusp midway between the ranula and the commisure. The mass was excised with sharp dissection. A second 2 × 3 mm mass was also observed on the underside of the leaflet and was excised in a similar fashion. Lesions were also observed at the commisure and on the left coronary cusp and were also excised. Pathologic examination subsequently revealed that these lesions were small fragments of myxoid tissue with hyalinization. A modified Maze procedure was also performed at the time of surgery and it required mobilization of the pulmonary veins bilaterally with application of radiofrequency lesions just proximal to the entrance of the left and right pulmonary veins. Another radiofrequency lesion was placed at the base of the left atrial appendage and this was then excised and then over sewn. Finally, the right atrium was then opened in a longitudinal fashion, the PFO was identified at the cephalad portion of the fossa ovalis and was then closed in a running fashion.

The patient was weaned from bypass without incident and a TEE performed in the operating room revealed no residual masses on the aortic valve or right to left shunt. However, a mild degree of tricuspid regurgitation was now identified for the first time. Both right ventricular size and systolic function remained normal. The patient was taken to the intensive care unit in stable condition and the immediate post-operative course was unremarkable. There was no need for either vasopressor or ionotropic support and the patient was extubated on the first post-operative day and subsequently transferred to a step down unit.

The patient did well until the night of the second post-operative day when she developed oliguria that progressively worsened followed by tachycardia and hypotension. At this time, a pronounced jugular venous distention was noted and a bedside transthoracic echo was immediately obtained to rule out the presence of pericardial tamponade. No pericardial effusion was noted; however, severe tricuspid regurgitation was now noted with dilatation and dysfunction of the right ventricle. In view of these findings, a spiral chest CT was requested but there was no evidence of pulmonary embolus; however, both superior as well as inferior vena cavae were dilated. The pericardium thickness was reported as normal. Because the clinical deterioration persisted, the patient was taken back to the operating room for mediastinal exploration; only a small amount of pericardial fluid was removed. Although the pericardial sac appeared to be under tension, no clot was found after extensive exploration.

**Figure 1:**
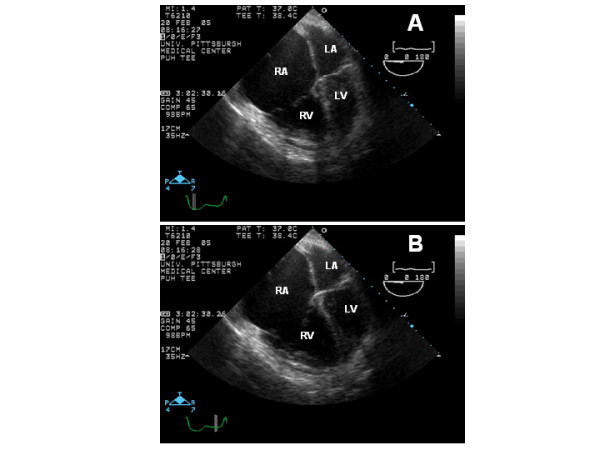
(A) An end-systolic mid esophageal 4-chamber view frame showing the markedly dilated right atria (RA) and ventricle (RV). The left atria (LA) and left ventricle (LV) are also seen in this picture. (B) An end-diastolic mid esophageal 4-chamber view frame showing the markedly dilated right atria (RA) and ventricle (RV). The left atria (LA) and left ventricle (LV) are also seen in this picture.

**Figure 2:**
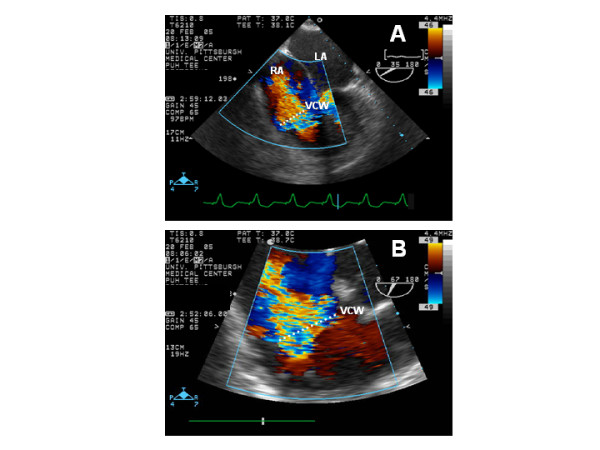
(A) An end-systolic mid esophageal 4-chamber view frame at 35 degrees shows the significant amount of tricuspid regurgitation with a large vena contracta width (VCW) as seen by the white dotted lines. (B) Close-up view at 67 degrees showing the severe degree of tricuspid regurgitation not only by color Doppler but by the large vena contracta width (VCW), as seen by the white dotted lines.

Despite a trial of medical management, the patient's cardiac index remained low and all facial and neck veins became progressively and markedly engorged. A repeated TEE demonstrated marked dilatation of the right-sided chambers with severe reduction in right ventricular systolic function (Figure 1) and severe tricuspid regurgitation as a result of an incomplete coaptation of the tricuspid valve leaflets (Figure 2). During this TEE, normal left ventricular contractility was noted with no regional wall motion abnormalities, with the exception of a paradoxical septal wall motion as expected post recent surgery, and normal left ventricular systolic function. In addition, pulsed interrogation of all four pulmonary veins showed normal velocities in order to rule out post-surgical Maze procedure pulmonary vein stenosis. No visible alteration in either the main or secondary pulmonary arteries was seen by TEE or CT. Although a clear unifying mechanism that would encompass the hemodynamic deterioration as well as the disproportionate symptoms of dyspnea with the rapidly progressive dilatation of all superficial neck and facial veins could not be given, it was clearly evident that the incomplete coaptation of the tricuspid valve leaflets, as seen on TEE, would certainly continue to further deteriorate the current clinical condition. Therefore, the patient was taken to the operating room again and upon opening the right atrium, the dilatation of the tricuspid valve annulus was confirmed resulting in failure of coaptation of the otherwise normal appearing tricuspid leaflets. Tricuspid annuloplasty with a 34 mm Cosgrove band was performed with marked improvement in cardiac output not requiring further inotropic support. A postoperative TEE revealed no evidence of residual tricuspid regurgitation and the chest was closed. The remainder of the patient's post-operative course was uncomplicated. Unfractionated intravenous heparin was started once the surgeon felt it was safe to maintain a therapeutic activated partial thromboplastin time. The pronounced engorgement of her neck and face veins resolved and she remained hemodynamically stable. After the 5^th ^postoperative day, the patient was transferred to a rehabilitation facility. At one-month follow-up, the patient has remained in atrial fibrillation, but is otherwise doing well.

## Conclusion

This case demonstrates an unusual sequence of post operative events that occurred after surgical resection of filamentous aortic valve masses, closure of a patent foramen ovale, and performance of modified Maze procedure for atrial fibrillation in a patient presenting with transient neurologic findings. The inconclusive nature and contradictory results of diagnostic testing made it difficult to accurately identify the responsible mechanism(s) and reminds us that in order to clearly solve any clinical mystery we have to review all possibilities, and despite the unclear nature of objective evidence, whatever remains, though improbable, must in the end make sense and indeed might be the most simple explanation.

Given the most obvious postoperative complication, we ruled out pericardial tamponade and then tried to make sense of the striking dilatation of all superficial neck and facial veins resembling superior vena cava syndrome [1-3]. The rapidly progressive nature of the venous engorgement was contrary to the known pathophysiology encountered by the clinical entities associated with superior vena cava syndrome that are rather insidious [3,4]. Furthermore, there was no recent or long-standing central catheter or pacer wire in place that would have incidentally caused venous thrombosis [5]. Therefore, additional considerations needed to be sought in order to explain this striking finding.

The possibility of embolization from one or more of the aortic valve masses, at the time of surgical resection, into the right coronary artery at the time of surgical resection, was also entertained as a possibility but quickly discarded given the lack of regional wall motion abnormalities in the left ventricle and the extremely unlikely chance of an isolated embolization to the right ventricular branch of the right coronary artery.

Closure of an atrial septal defect in a patient with significant pulmonary hypertension might cause some of the hemodynamic derangements seen in this patient; however, closure of a PFO with similar consequences would be mostly unlikely.

Mobilization of all pulmonary veins with application of radiofrequency lesions just proximal to their entrance may account for all the findings in our case [6-8], however, stenosis of the pulmonary veins are usually noted at a much later time and would not resolve with tricuspid valve repair.

Lastly, manual damage to the tricuspid valve during surgical manipulation has to be taken into account; however, the valve anatomy was intact and only the annulus was dilated. So in the end, pulmonary embolism (PE) remained as the most likely possibility despite the conflicting diagnostic testing results. Although helical (spiral) CT with bilateral upper extremity contrast injection has a reported sensitivity of 98 percent for diagnosing PE [9]; in some studies the sensitivities have ranged from 53 to 87 percent, even for segmental or larger emboli [10-12]. Obviously, the specificity of helical CT scanning depends on reader experience and results must be interpreted with caution, particularly if the clinical probability of pulmonary emboli and the CT results are discordant. A ventilation-perfusion scan was not considered in this case given the patient's critical and unstable condition that would place the patient at an enormous risk to undergo this test. Furthermore, the results of a recent report that demonstrated that helical CT had a greater discriminatory power than ventilation-perfusion scanning with normal and/or near-normal threshold to exclude pulmonary embolism we decided not to pursue this venue, but instead consider another diagnostic modality. [13]

It has to mentioned that the use of D-dimer in this particular case was considered of limited value given the known altered fibrinolysis and platelet function that occurs in patients post cardiopulmonary bypass resulting in an increase in soluble fibrin, fibrinogen degradation products and D-dimer. [14,15]

**Figure 3:**
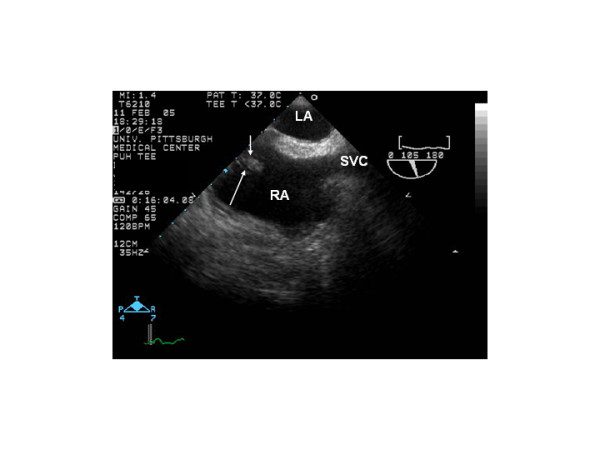
Bi-caval transesophageal view showing the RA and LA divided by the interatrial septum. In this view the superior vena cava (SVC) is also seen. Please note, the small homogeneous mass arising from the orifice of the inferior vena cava, as seen flanked by the arrows.

However, of particular importance and relevance to this discussion are the findings noted on echocardiography. It has been suggested that in up to more than 80 percent of patients with PE, some echocardiographic abnormality that includes either right ventricular dilatation and dysfunction or color Doppler manifestation of tricuspid regurgitation will be present in these patients using this diagnostic modality [16,17]. Thus, although we should have discarded PE on our case based on the spiral chest CT results, we continued pursuing this diagnostic entity given the unexplained dilatation of both inferior and superior vena cavae [18]. In fact, upon careful review of the post operative TEE, a small homogeneous echodensity arising from the inferior vena cava, as seen in Figure 3, was seen. This homogeneous echodensity, highly suggestive of a clot, that probably formed during closure of the PFO or less likely present on the original study; but also undetected, then migrated to the right ventricle and eventually to the main pulmonary artery at the termination of the surgical procedure and it was not responsible for the clinical events until later in the postoperative course.

Although the diagnosis of PE in this case will certainly account for the clinical findings and hemodynamic abnormalities, we still have to explain the degree of tricuspid regurgitation and the tricuspid annular dilatation noted post operatively. It has been documented, that chronic thromboembolic pulmonary hypertension is associated with right ventricular dilatation, high right-sided filling pressures, and functional tricuspid regurgitation. Although we were not able to obtain a copy of the original study, it is possible that some degree of right ventricular dysfunction was already present and was further compounded by an acute event that disrupted homeostasis and resulted in the abnormal hemodynamics. This is a likely possibility, since it has been postulated, that the tricuspid regurgitation as a result of chronic thromboembolic disease that produces pulmonary hypertension is caused by tricuspid annular dilatation with displacement of the papillary muscles [19]. In addition, as reported by Rosenberger and associates, TEE evidence of right ventricular dysfunction and new or worsening degree of tricuspid regurgitation are found in a significant number of patients with PE even when direct visualization of a clot was not found on TEE or it was questionable. [20] In this case, leftward interatrial septal bowing could not be used as a TEE finding since the patient was intubated and mechanically ventilated.

Finally, the decision of proceeding with tricuspid valve repair was basically made given the TEE results showing incomplete coaptation of the tricuspid valve leaflets with a clearly dysfunctional right ventricle in a patient that continue to deteriorate despite inotrope support. In a recent report by Dreyfus and associates, remodeling annuloplasty of the tricuspid valve based on tricuspid dilation has shown to improve functional status irrespective of the grade of regurgitation and these authors conclude that if left alone, tricuspid dilatation is an ongoing disease process that will, with time, lead to severe tricuspid regurgitation [21] and this in turn will continue to compromise right ventricular hemodynamics.

### Conclusion

This case demonstrates an unusual post operative course of events and physical findings in which a unifying diagnosis was difficult to identify given the conflicting nature of diagnostic testing. We present a careful retrospective examination of all clinical data and review possible clinical entities that could have been implicated in this particular case. In addition, we point out how TEE findings were most useful in identifying the best course of action. We postulate that in our case, resolution of the PE with anticoagulation [22] and reestablishment of a normal right ventricular geometry with tricuspid valve repair [23] worked in unison in restoring normal hemodynamics and resolving both dyspnea and venous dilatation.

## Abbreviations

TTE Transthoracic echocardiography

TEE Transesophageal echocardiography

PE Pulmonary embolism

PFO Patent foramen ovale

CT Computed tomography

RA Right atria

LA Left atria

RV Right ventricle

LV Left ventricle

VCW Vena contracta width

SVC Superior vena cava

## Competing interests

The author(s) declare that they have no competing interests.

## Authors' contributions

Dr. Pellegrini performed the surgery and Dr. Kaczorowski assisted. Dr. López-Candales performed and interpreted the TTE and TEE. Dr. Kaczorowski and Dr. López-Candales prepared the manuscript and reviewed the literauture. All the authors have approved the final review of the manucsript.
